# Systemic Delivery of scAAV8-Encoded MiR-29a Ameliorates Hepatic Fibrosis in Carbon Tetrachloride-Treated Mice

**DOI:** 10.1371/journal.pone.0124411

**Published:** 2015-04-29

**Authors:** Matthew K. Knabel, Kalyani Ramachandran, Sunil Karhadkar, Hun-Way Hwang, Tyler J. Creamer, Raghu R. Chivukula, Farooq Sheikh, K. Reed Clark, Michael Torbenson, Robert A. Montgomery, Andrew M. Cameron, Joshua T. Mendell, Daniel S. Warren

**Affiliations:** 1 Department of Surgery, Johns Hopkins University School of Medicine, Baltimore, Maryland, United States of America; 2 The McKusick-Nathans Institute of Genetic Medicine, Johns Hopkins University School of Medicine, Baltimore, Maryland, United States of America; 3 Department of Surgery, Temple University School of Medicine, Philadelphia, PA, United States of America; 4 Laboratory of Molecular Neuro-oncology, Rockefeller University, New York, New York, United States of America; 5 Department of Medicine, Massachusetts General Hospital, Boston, Massachusetts, United States of America; 6 Department of Cardiology, Washington Hospital Center, Washington, DC, United States of America; 7 Center for Gene Therapy, The Research Institute at Nationwide Children’s Hospital, Columbus, Ohio, United States of America; 8 Department of Pathology, Mayo Clinic, Rochester, Minnesota, United States of America; 9 Department of Molecular Biology, UT Southwestern Medical Center, Dallas, Texas, United States of America; 10 Center for Regenerative Science and Medicine, UT Southwestern Medical Center, Dallas, Texas, United States of America; 11 Simmons Cancer Center, UT Southwestern Medical Center, Dallas, Texas, United States of America; University of Navarra School of Medicine and Center for Applied Medical Research (CIMA), SPAIN

## Abstract

Fibrosis refers to the accumulation of excess extracellular matrix (ECM) components and represents a key feature of many chronic inflammatory diseases. Unfortunately, no currently available treatments specifically target this important pathogenic mechanism. MicroRNAs (miRNAs) are short, non-coding RNAs that post-transcriptionally repress target gene expression and the development of miRNA-based therapeutics is being actively pursued for a diverse array of diseases. Because a single miRNA can target multiple genes, often within the same pathway, variations in the level of individual miRNAs can potently influence disease phenotypes. Members of the miR-29 family, which include miR-29a, miR-29b and miR-29c, are strong inhibitors of ECM synthesis and fibrosis-associated decreases in miR-29 have been reported in multiple organs. We observed downregulation of miR-29a/b/c in fibrotic livers of carbon tetrachloride (CCl_4_) treated mice as well as in isolated human hepatocytes exposed to the pro-fibrotic cytokine TGF-β. Importantly, we demonstrate that a single systemic injection of a miR-29a expressing adeno-associated virus (AAV) can prevent and even reverse histologic and biochemical evidence of fibrosis despite continued exposure to CCl_4_. The observed therapeutic benefits were associated with AAV transduction of hepatocytes but not hepatic stellate cells, which are the main ECM producing cells in fibroproliferative liver diseases. Our data therefore demonstrate that delivery of miR-29 to the hepatic parenchyma using a clinically relevant gene delivery platform protects injured livers against fibrosis and, given the consistent fibrosis-associated downregulation of miR-29, suggests AAV-miR-29 based therapies may be effective in treating a variety of fibroproliferative disorders.

## Introduction

Acute tissue injury is characterized by transient increases in inflammation and extracellular matrix (ECM) that resolve over time as the wound heals and homeostatic tissue remodeling returns matrix proteins and local cellular populations to pre-injury levels. In contrast, many chronic inflammatory stimuli including infection, autoimmunity and toxin exposure are associated with persistently elevated myofibroblast populations and unabated matrix synthesis and deposition. The consequential accumulation of excess ECM, commonly referred to as fibrosis, displaces functional parenchyma and contributes to organ dysfunction and failure. Fibrosis can occur in all tissues of the body and is a central pathological component of diseases that affect the heart, liver, lungs and kidneys. Unfortunately, and despite significant progress in our understanding of fibroproliferative pathways, organ fibrosis continues to account for a significant fraction of the morbidity and mortality in the developed world with few, if any, effective treatments [1].

The ECM not only provides critical structural support for tissues but also establishes a dynamic microenvironment that influences the proliferation, migration and function of surrounding cells. Regulating the composition and abundance of matrix proteins is thus an important biological process and recent studies have identified microRNAs (miRNAs) as key regulators of several ECM structural proteins as well as the cytokines and proteases that regulate their synthesis, deposition and stability (reviewed in [2–4]). MicroRNAs are short, non-coding RNAs that bind to partially complementary sites in the 3’UTR of target messenger RNAs (mRNAs) and post-transcriptionally repress their expression. Aberrant regulation of miRNAs has been implicated in the pathogenesis of many human diseases [5,6] and therapeutic approaches that seek to normalize the expression of dysregulated miRNAs could potentially be applied to a wide array of disorders [7]. To that end, antisense oligonucleotides or “sponges” (synthetic concatemers of miRNA target sites) can be used to inhibit overexpressed miRNAs while synthetic mimics or ectopic expression of miRNA precursors can functionally replace repressed miRNAs [8,9]. Although the lack of established methods for targeted delivery to specific tissues or cell-types remains a significant hurdle, the small size and relative stability of mature miRNAs represent inherent advantages compared to other nucleic acid based therapeutic strategies. In addition, while the therapeutic threshold will vary for different miRNAs and conditions, the pleiotropic nature of miRNA regulation suggests that even partial normalization of a dysregulated miRNA could provide significant therapeutic benefit.

Numerous extracellular matrix (ECM) proteins including several collagens, elastin and fibrillin are validated targets of the miR-29 family [10–15], which includes miR-29a, miR-29b and miR-29c. In humans and mice these miRNAs are encoded by two distinct transcripts (miR-29a/miR-29b-1 and miR-29b-2/miR-29c) and fibrosis-associated decreases in mature miR-29 levels have been reported in diverse tissues [10,16–22]. Moreover, it has been demonstrated that adenovirus-mediated expression of miR-29a can attenuate carbon tetrachloride (CCl_4_)-induced liver fibrosis in mice [23]. Nevertheless, use of a clinically relevant delivery system to restore hepatic miR-29 expression and reverse existing liver fibrosis, the likely clinical scenario in which this therapy would be implemented, has not yet been demonstrated. Adeno-associated viral vectors (AAV) are currently being tested in several clinical trials [24] and we show here that systemic administration of AAV-miR-29a strongly prevents and reverses hepatic fibrosis in carbon tetrachloride (CCl_4_)-treated mice. Surprisingly, these therapeutic responses were associated with AAV transduction of hepatocytes but not hepatic stellate cells, which are the main ECM producing cells in fibroproliferative liver diseases. Our findings highlight the potential of clinically viable miR-29-based therapies for treating established organ fibrosis in chronically injured tissues.

## Materials and Methods

### AAV Vector Construction

scAAV.miR29a.eGFP was constructed by amplifying miR-29a from human genomic DNA using the following primers: 5'- ATACCGGGCCGGCCGAGCCCAATGTATGCTGGAT-3' (forward) and 5'- ATACCGGGCCGGCCTGCATTATTGCTTTGCATTTG-3' (reverse). The amplicon was cloned into the FseI site of scAAV.eGFP [25].

### Carbon Tetrachloride (CCl_4_) Treatment and Vector delivery

**Fig 1:**
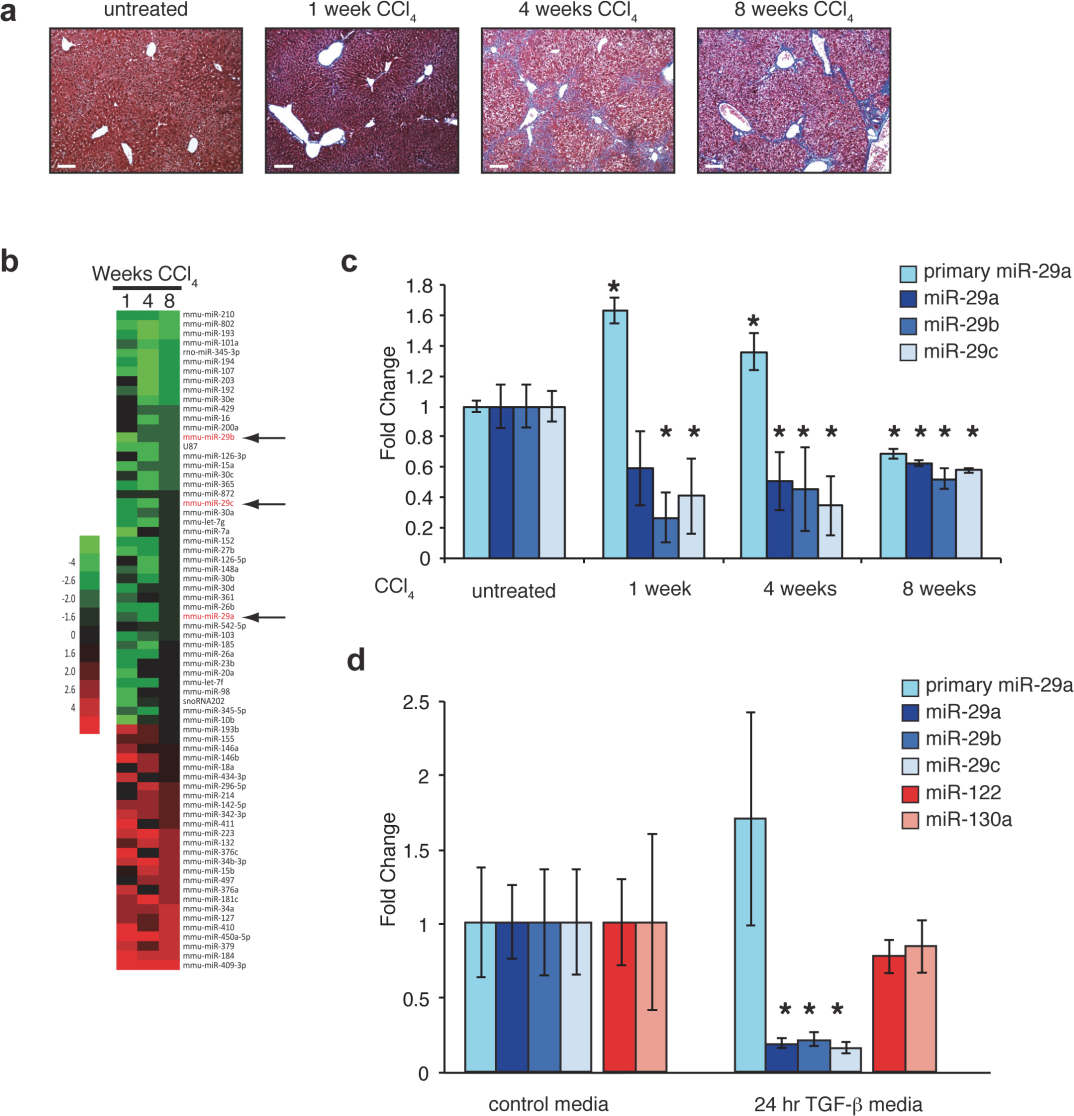
Primary and mature miR-29 expression levels in murine liver and isolated human hepatocytes. (a) Carbon tetrachloride-mediated liver fibrosis. Trichrome-stained liver sections demonstrating progressive fibrosis during 8 weeks of CCl_4_ exposure. Scale bar = 100μm (b) Heat map of miRNA expression levels after 1, 4, and 8 weeks of CCl_4_ exposure (compared to normal liver). The top 70 miRNAs with the largest average fold change are shown and sorted by fold change at 8 weeks. miR-29 family members indicated with arrows. (c) Hepatic expression levels of primary and mature miR-29a,b,c in CCl_4_-treated mice. Average fold changes for 1 week (n = 4), 4 week (n = 3) and 8 week (n = 2) treatment groups were calculated using normal liver (no CCl_4_) as a reference (n = 3). Error bars represent +/- one standard deviation. (* = p<0.05 compared to normal liver). (d) TGF-β represses miR-29 expression in human hepatocytes. Average fold change of primary and mature miR-29a/b/c as well as miR-122 and miR-130a in TGF-β treated hepatocytes were calculated using control media-treated cells as a reference. Error bars represent +/- one standard deviation. (* = p<0.05 compared to control media).

**Fig 2:**
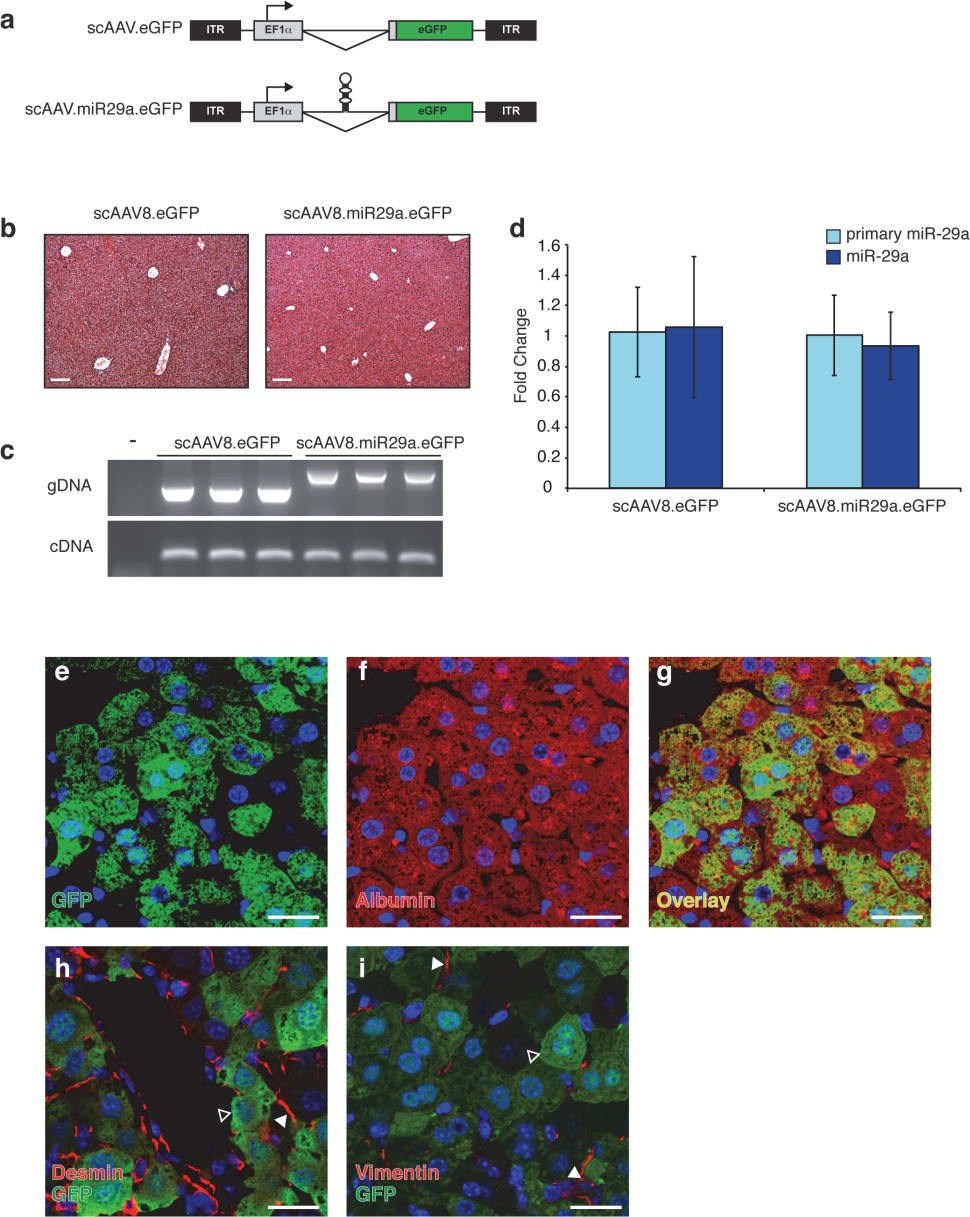
scAAV8 transduction and miR-29 expression levels in murine liver. (a) Schematic representation of scAAV vectors depicting locations of inverted terminal repeats (ITRs), elongation factor 1-alpha promoter (EF1α), miRNA (shown in hairpin form), and enhanced green fluorescent protein (eGFP) open reading frame. (b) Transduction with scAAV8 does not disrupt normal liver architecture. Trichrome stained liver sections from AAV-transduced animals demonstrating normal histology. Scale bar = 100μm (c) Viral genomic DNA (gDNA) and mRNA from the EF1α transcription unit (cDNA) are readily detectable in mouse liver following transduction with 2x10^11^ vg of scAAV8.eGFP (n = 3) or scAAV8.miR29a.eGFP (n = 3). The presence of the hairpin accounts for the increased size of the scAAV8.miR29a.eGFP gDNA amplicon. (d) Hepatic expression of primary and mature miR-29a in scAAV8 transduced mice. Average fold change for each treatment group was calculated using scAAV8.eGFP treated mice as a reference (n = 3). Error bars represent +/- one standard deviation. (e-i) Localization of AAV-mediated GFP expression in transduced mouse liver. Sections of transduced livers were co-immunostained for GFP (e and g-i; green) and markers of hepatocytes (Albumin f and g; red) or stellate cells (Desmin h; Vimentin i; red). Open arrowheads indicate GFP+ hepatocyte and filled arrowheads indicate desmin+ or vimentin+ stellate cells. All sections were counterstained with Hoechst (blue). Confocal images were captured with a 40x objective and are shown at 2x zoom. Scale bar = 20μm.

**Fig 3:**
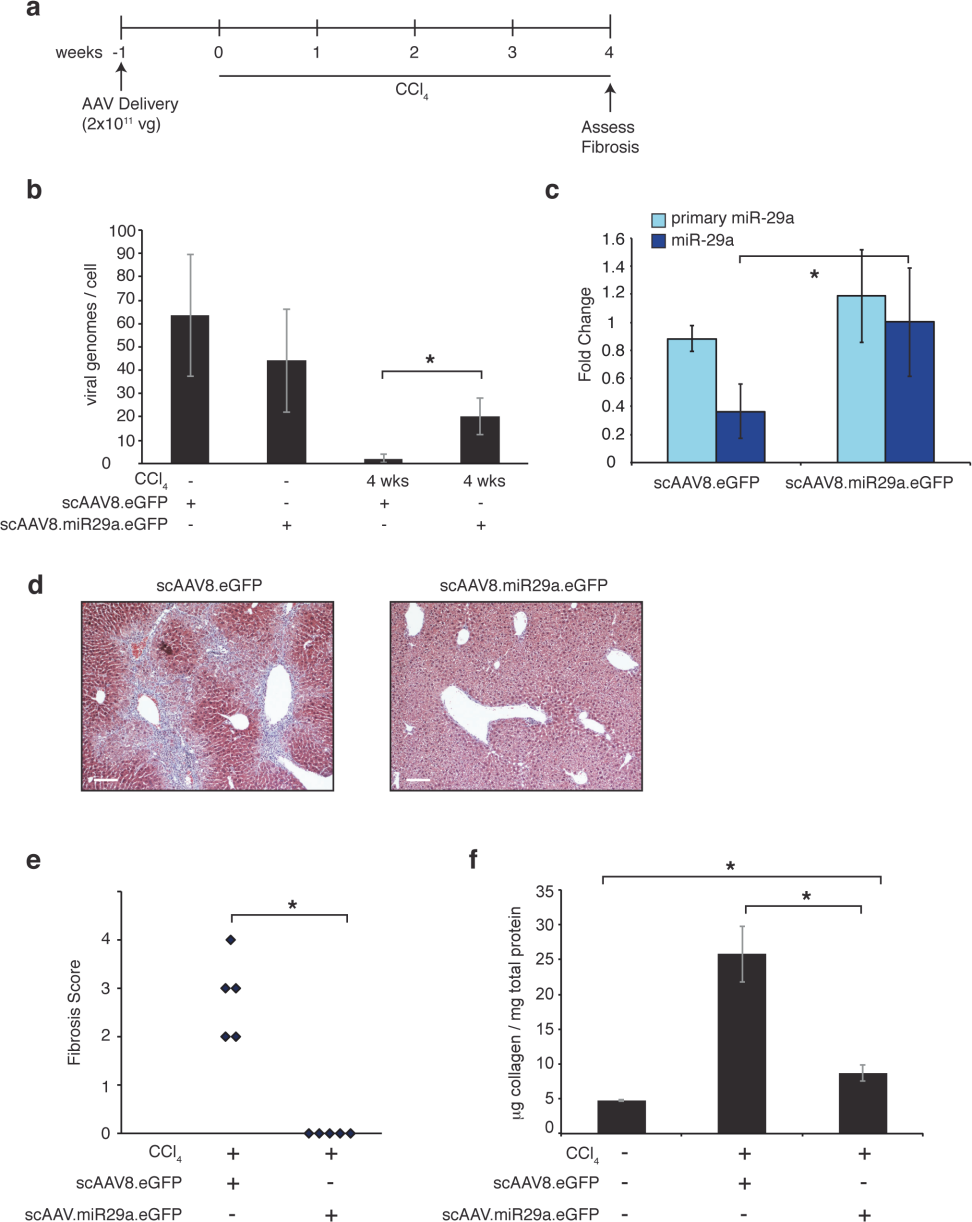
Pre-treatment with scAAV8.miR29a.eGFP prevents CCl_4_-mediated hepatic fibrosis. (a) Timeline of AAV delivery and CCl_4_ treatment. (b) Estimate of viral genomes/cell in livers of scAAV-transduced mice. A portion of the viral genome (GFP) and a non-repetitive locus in the mouse genome (DGCR8) were separately amplified from whole liver genomic DNA. Standard curves of known amounts of AAV8.eGFP plasmid DNA and whole liver genomic DNA were used to determine the number of viral and cellular genomes in each sample, respectively. (c) Hepatic expression of primary and mature miR-29a in the livers of CCl_4_-treated scAAV8.eGFP (n = 5) and scAAV8.miR29a.eGFP transduced animals (n = 5). Average fold change for each treatment group was calculated using normal (no CCl_4_) scAAV8.eGFP treated mice as a reference (n = 3). Error bars represent +/- one standard deviation. (d) Trichrome staining reveals reduced collagen deposition (blue) in scAAV8.miR29a.eGFP transduced livers. Scale bar = 100μm (e) The degree of fibrosis was scored on a scale of 0–4 by a trained pathologist (blinded to treatment condition) and the score for each individual animal is shown. (f) Quantitative determination of hepatic collagen levels in transduced animals. Error bars represent +/- one standard deviation. (* = p<0.05).

**Fig 4:**
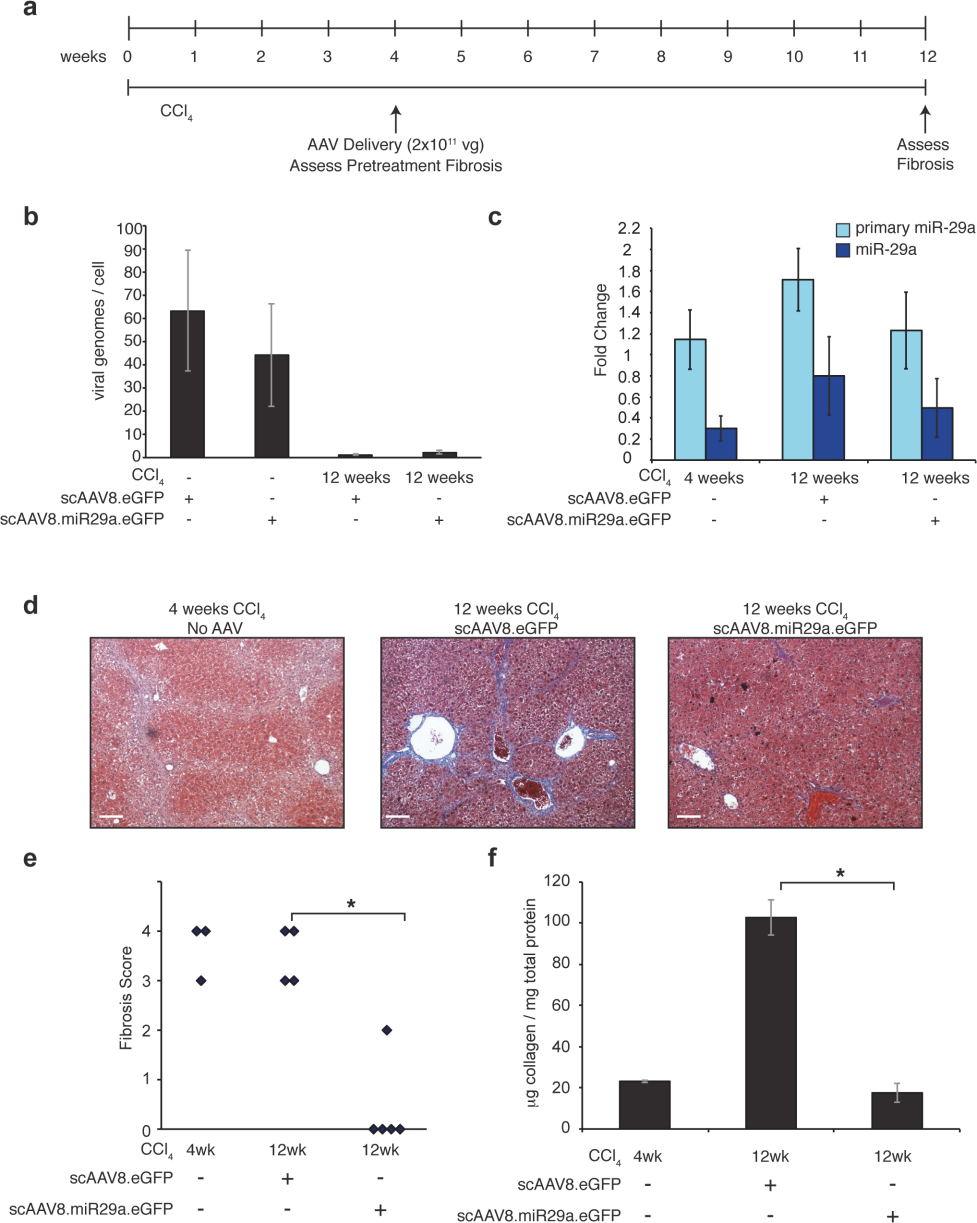
Intervention with scAAV8.miR29a.eGFP reverses histologic evidence of CCl_4_-mediated hepatic fibrosis. (a) Timeline of AAV delivery and CCl_4_ treatment. (b) Estimate of viral genomes/cell in livers of scAAV-transduced mice. A portion of the viral genome (GFP) and a non-repetitive locus in the mouse genome (DGCR8) were separately amplified from whole liver genomic DNA. Standard curves of known amounts of AAV8.eGFP plasmid DNA and whole liver genomic DNA were used to determine the number of viral and cellular genomes in each sample, respectively. (c) Hepatic expression of primary and mature miR-29a in CCl_4_-treated scAAV8.eGFP (n = 4; one of the five scAAV8 injected mice died during CCl_4_ treatment) and scAAV8.miR29a.eGFP transduced animals (n = 5). In parallel, three additional animals were sacrificed after 4 weeks of CCl_4_ treatment to establish the level of fibrosis present at the time of viral delivery (pre-treatment). Average fold change for each treatment group was calculated using normal (no CCl_4_) scAAV8.eGFP treated mice as a reference (n = 3). Error bars represent +/- one standard deviation. (d) Trichrome staining reveals reduced collagen deposition (blue) in scAAV8.miR29a.eGFP transduced livers compared to either pre-treatment (4 weeks CCl_4_) or scAAV8.eGFP treatment (12 weeks CCl_4_). Scale bar = 100μm (e) The degree of fibrosis was scored on a scale of 0–4 by a trained pathologist (blinded to treatment condition) and the score for each individual animal is shown. (f) Quantitative determination of hepatic collagen levels in transduced animals. Error bars represent +/- one standard deviation. (* = p<0.05).

C57/BL6 mice received intraperitoneal injections of 1 ml/kg carbon tetrachloride (Sigma-Aldrich) diluted 1:7 in corn oil twice a week for up to 12 weeks. AAV was administered at a dose of 2x10^11^ viral genomes (vg) per animal (Figs 1, 2, 3 and 4) or 1x10^12^ vg/animal (high dose; S2 Fig only) via tail vein injection with a 30g needle. The Animal Care and Use Committee of the Johns Hopkins University School of Medicine reviewed and approved this study (Protocol MO13M227) and all housing and procedures were carried out in strict accordance with their policies and recommendations.

### RNA isolation and PCR

Total RNA was isolated from cultured cells or whole liver tissue using Trizol (Invitrogen) and treated with DNase I (Invitrogen) according to the manufacturers’ protocols. Expression of 18s rRNA, primary miR-29a, mature miR-29a/b/c, miR-122 and miR-130a was assessed using individual Taqman assays (Applied Biosystems). Non-quantitative amplification of viral gDNA and cDNA was performed using DreamTaq Green Master Mix (Fermentas) according to manufacturer’s protocol using the following primers: 5'-CGCAACGGGTTTGCCGCCAGAAC-3' (forward); 5'-GGCCGTTTACGTCGCCGTCCAG-3' (reverse).

### MicroRNA Array

Total liver RNA was prepared using a mirVana miRNA Isolation Kit (Ambion) according to the manufacturer’s protocol. RNA (500ng) was reverse transcribed without pre-amplification using Megaplex RT Primers Rodent Pool A v2.0 (Applied Biosystems) and a TaqMan MicroRNA Reverse Transcription Kit (Applied Biosystems), according to the manufacturer’s protocol. The Taqman array was run on the 7900HT Fast Real-Time PCR System with TaqMan Array Rodent MicroRNA A Cards v2.0 (Applied Biosystems), according to the manufacturer’s protocol. The geometric mean of each plate was used for normalization and the 70 miRNAs with the greatest average fold change across 1, 4, and 8 weeks of CCl_4_ treatment are presented in the heat map (Fig 1). The results of the Taqman array are available through the NCBI Gene Expression Omnibus (Accesssion # GSE66278; www.ncbi.nlm.nih.gov/geo/).

### Immunostaining

Formalin-fixed, paraffin-embedded tissues were sectioned (5μm thickness), transferred to Superfrost/Plus Microscope Slides (Fisher Scientific), and incubated for 30’ at 60°C. Slides were washed with PBST and blocked with PBS + 5% fetal bovine serum (Sigma-Aldrich) and 3% goat serum (Sigma-Aldrich). Slides were then incubated for 1 hour at room temperature with antibodies specific for albumin (Santa Cruz Biotechnologies), vimentin (Millipore), desmin (Sigma-Aldrich), α-SMA (Sigma-Aldrich), or GFP (Invitrogen). They were then washed in PBST and incubated with combinations of the following secondary antibodies: Cy3 labeled goat anti-rabbit IgG (GE Healthcare), Cy3 labeled anti mouse and Cy3 anti-chicken (Millipore), or Alexa Fluor 488 anti-rabbit (Cell Signaling Technology). After washing in PBST, slides were counterstained with Hoechst 33258 (Molecular Probes) and mounted using Prolong Gold Antifade Reagent (Invitrogen).

### Collagen Assay

Collagen levels were determined by Sircol Soluble Collagen Assay (Biocolor), which was performed according to the manufacturer’s protocol.

### Histology and Fibrosis Scoring

Sections of formalin fixed, paraffin embedded liver samples were stained with hematoxylin and eosin as well as Masson’s trichrome by the Johns Hopkins Reference Histology Lab (Baltimore, MD). A trained pathologist, who was blinded to AAV and CCl_4_ treatment details, scored the hepatic fibrosis of each animal on a scale of 0–4.

### Quantification of Viral Genomes

A portion of the viral genome (5’-CCACTACCTGAGCACCCAGTC-3’ (forward); 5’-TCCAGCAGGACCATGTGATC-3’ (reverse)) and a non-repetitive locus in the mouse genome (DGCR8; 5’-CCATCAGGCAATGGCTCTGT-3’ (forward); 5’-TGCAGGATGTTTTTTGTGTTCTG-3’ (reverse)) were separately amplified from whole liver genomic DNA samples from transduced mice. Standard curves of known amounts of AAV8.eGFP plasmid DNA (2.96x10^-6^ pg per copy) and whole liver genomic DNA (5.8 pg dsDNA per diploid cellular genome) were used to determine the number of viral and cellular genomes in each sample.

### *In vitro* TGF-beta treatment of Human Hepatocytes

Human hepatocytes (CellzDirect) were plated on dishes coated with 5μg/ml collagen (Gibco) in serum-rich media [DMEM +10% FBS, 15mM Hepes, 10μg/ml gentamycin (Quality Biological), 1x ITS (Sigma-Aldrich), 1mM dexamethasone (Sigma-Aldrich), and 2mM L-glutamine (Quality Biological)]. Twenty-four hours later, the cells were washed and the media was replaced with serum-free media. After 24h the cells were washed and fresh serum-free media with or without 5ng/ml TGF-β (Roche) was added. RNA was isolated 24 hours after the addition of TGF-β.

### Statistics

All statistical comparisons of qPCR data were performed using REST 2009 software (Qiagen). A two-tailed T-test was used to calculate p values for comparisons of fibrosis scores and quantitative collagen assays.

## Results

### miR-29, a potent regulator of ECM production, is down regulated in fibrotic livers and TGF-β treated hepatocytes

To identify miRNAs that potentially regulate fibroproliferative processes, we profiled miRNA expression in the livers of mice exposed to carbon tetrachloride (CCl_4_). This widely-used model of hepatic fibrosis is characterized by progressive increases in collagen deposition throughout the period of exposure to CCl_4_ (Fig 1A). Consistent with prior reports [21,23], we observed downregulation of miRNAs belonging to the miR-29 family (miR-29a, miR-29b, and miR-29c) among a larger set of dysregulated miRNAs in the livers of mice treated with CCl_4_ for up to eight weeks (Fig 1B and 1C). The primary transcript of miR-29a was significantly increased after 1 and 4 weeks of CCl_4_ exposure suggesting that altered processing and/or decreased stability of the mature miRNA contributes to the observed reduction in mature miR-29 (Fig 1C).

The fibrosis-associated downregulation of miR-29 is of particular interest because these miRNAs target the transcripts of a large number of ECM proteins including several collagens, elastin, and fibrillin. Mutating the binding sites or inhibiting endogenous miR-29 with antisense oligonucleotides de-repressed a *COL1A1* 3' UTR luciferase reporter construct upon transfection into primary fibroblasts (S1 Fig). Antisense-mediated inhibition of miR-29 also strongly de-repressed endogenous type I collagen expression (S1 Fig).

Activated stellate cells and their derivatives are responsible for most if not all of the ECM production in liver fibrosis and previous studies have shown that inflammatory stimuli decrease miR-29 levels in purified stellate cells [21]. We determined that miR-29 family members are also expressed in purified human hepatocytes and that stimulation of hepatocytes with TGF-β, a potent fibroproliferative cytokine, resulted in decreased mature miR-29a/b/c without a significant reduction in pri-miR-29a (Fig 1D), similar to the pattern observed in liver samples of CCl_4_ treated mice.

### Hepatocyte restricted transgene expression following systemic delivery of scAAV8-miR29a

To facilitate therapeutic delivery of miR-29 to injured livers we adapted a previously described AAV vector system [25]. This self-complementary AAV vector (scAAV.eGFP) contains enhanced green fluorescent protein (eGFP) driven by the ubiquitously expressed elongation factor 1 alpha (EF1α) promoter (Fig 2A). To facilitate the simultaneous production of miR-29a and eGFP from a single transcript, we created scAAV.miR-29a.eGFP by cloning miR29a into the short intron that is contained in the EF1α promoter unit [26]. Transient transfection of HEK293 cells with increasing amounts of scAAV.miR29a.eGFP plasmid was associated with concordant increases in the level of miR-29a (S2 Fig). For *in vivo* delivery, scAAV.eGFP or scAAV.miR29a.eGFP were packaged in AAV serotype 8 capsids, which are known to efficiently transduce liver. Four weeks after a single tail vein injection of 2×10^11^ vector genomes (vg), the livers of both scAAV.eGFP or scAAV.miR29a.eGFP treated mice were histologically normal (Fig 2B) and viral genomic DNA and transgene expression (GFP) were readily detectable in liver samples (Fig 2C). Evidence of extra-hepatic GFP expression was not observed in the heart, lungs, small intestine, kidney, or spleen (data not shown).

To identify which cells within the liver were expressing AAV encoded transgenes, sections from injected mice were stained with antibodies against GFP and markers of specific cell types including albumin (hepatocytes), desmin (stellate cells) and vimentin (stellate cells). GFP expression consistently co-localized with albumin^+^ hepatocytes but no evidence of transgene expression in desmin^+^ or vimentin^+^ cells was observed (Fig 2E–2I). Surprisingly, despite documented hepatocyte AAV transduction, no increase in hepatic (Fig 2D) or serum (data not shown) levels of mature miR-29a was observed in scAAV8-miR-29a.eGFP mice compared to scAAV8.eGFP injected mice. Raising the dose of AAV five fold to 1X10^12^ vg also failed to increase miR-29a above endogenous levels (S2 Fig), even though the equivalent dose of a miR-26a-expressing scAAV8 resulted in significant overexpression of miR-26 [25].

### Pretreatment with scAAV8.miR29a.eGFP protects mice from fibrotic injury

To determine if AAV-mediated miR-29a delivery could prevent fibrosis, mice were given a single tail-vein injection of 2x10^11^ vg of either scAAV8.eGFP (n = 5) or scAAV8.miR29a.eGFP (n = 5) one week prior to initiation of a 4-week course of CCl_4_ treatment (Fig 3A). At the endpoint of the experiment, the hepatotoxic effects of CCl_4_ were associated with reduced viral copy number in the livers of all treated animals compared to uninjured mice, however miR-29 treated mice exhibited a much smaller reduction than mice receiving control virus (Fig 3B). After 4 weeks of CCl_4_, scAAV8.eGFP treated mice were characterized by reduced miR-29a expression and significant increases in histological and biochemical measures of fibrosis (Fig 3C–3F). In contrast, scAAV8.miR29a.eGFP treated mice exhibited normal hepatic miR-29 expression (Fig 3C), lacked any histologic evidence of fibrosis (Fig 3D and 3E) and had only a slight increase in total collagen (Fig 3F). ALT/AST levels increased similarly after CCl_4_ treatment in both the scAAV8.miR29.eGFP-treated and control mice (S3 Fig), indicating equivalent liver injury in both cohorts. Nevertheless, scAAV8.eGFP.miR29a treatment was associated with reduced immunostaining for α-SMA (S4 Fig), suggesting reduced activation of stellate cells and myofibroblasts. Together, these observations suggest that administration of scAAV8.miR29a.eGFP was sufficient to maintain normal miR-29 levels and thereby block *de novo* fibrosis in the setting of chronic liver injury.

### A single injection of scAAV8.miR29a.eGFP reverses histologic evidence of fibrosis in mice despite ongoing treatment with CCl_4_

Having demonstrated that pre-treated mice are effectively protected from fibrosis, we sought to determine if scAAV8.miR29a.eGFP could halt or reverse fibrosis when delivered in the context of established disease. Thus, we evaluated a second experimental design in which mice received a single injection of virus after completing four weeks of a 12 week course of CCl_4_ treatment (Fig 4A). Consistent with our earlier observations, mice that received scAAV8.miR29a.eGFP (n = 5) had significantly lower fibrosis than scAAV8.eGFP-treated mice (n = 4; 1 of 5 injected animals died before reaching 12 weeks of CCl_4_ treatment) (Fig 4D–4F). Moreover, blinded histopathologic scoring revealed that miR-29a-treated mice had even less evidence of fibrosis than was present at the time of AAV injection, indicating that therapeutic delivery of miR-29a resulted in reversal of established fibrosis despite the continued administration of CCl_4_. Liver injury and associated hepatocyte proliferation are known to rapidly dilute AAV vector genomes [27] and 8 weeks after injection (12 weeks total CCl_4_) viral genomes were very low in both control and miR-29 treated animals (Fig 4B) and miR-29a expression was repressed in both scAAV8.eGFP and scAAV8.miR29a.eGFP treatment groups (Fig 4C). Thus, a single injection of 2x10^11^ scAAV8.miR-29a.eGFP genomes is sufficient to normalize hepatic miR-29a expression under sustained CCl_4_ treatment for at least 4 weeks (Fig 3C) but less than 8 weeks. However, GFP protein was readily detectable in a high percentage of hepatocytes in scAAV8.miR-29a.eGFP mice (S5 Fig) at 12 weeks, suggesting that transgene expression, and therefore virally produced miR-29a, persisted throughout most of the 8 weeks of CCl4 exposure.

## Discussion

ECM synthesis and deposition is the final common pathway of all fibrotic disorders and therapeutic strategies that target this process would be highly attractive. miR-29 family members have been shown to inhibit the synthesis of collagen and other important ECM proteins and the anti-fibrotic effects of miR-29 expression in multiple tissues including liver, lung, heart, and muscle have been demonstrated [10,23,28–30]. Here we report that AAV-mediated restoration of miR-29 expression in a mouse model of liver fibrosis provides significant anti-fibrotic protection. Pre-treatment with a single injection of scAAV8.eGFP.miR29a completely prevented the development of fibrosis during 4 weeks of CCl_4_ exposure (Fig 3). In a second more clinically relevant model, we further demonstrate that intervention with a single injection of scAAV8.eGFP.miR29a after four weeks of a 12-week course of CCl_4_ results in partial to complete regression of the pre-existing fibrosis (Fig 4). AAV vectors are being used in several clinical trials [24] and our data provides the first evidence that a clinically relevant miR-29 delivery platform can reverse established liver fibrosis.

The AAV serotype 8 virions used in this study have been shown to efficiently transduce hepatocytes [31,32] and we observed that a single injection of 2x10^11^ viral genomes was associated with detectable GFP expression in about 50% of albumin^+^ hepatocytes. However, we did not find any evidence of GFP transgene expression in vimentin^+^ or desmin^+^ stellate cell populations (Fig 2E–2I). This is significant because activated stellate cells and their derivatives are responsible for most if not all of the ECM production in liver fibrosis and restoration of normal miR-29 expression in activated stellate cells could repress ongoing ECM protein synthesis and thus provide significant anti-fibrotic protection. However, the lack of detectable scAAV8.eGFP.miR29a transgene expression in stellate cells suggests additional mechanisms likely contribute to the observed protection against CCl_4_-induced liver fibrosis. Several lines of evidence support the possibility that transgene-derived miR-29a could directly or indirectly inhibit the production of profibrotic cytokines or metabolic intermediates in hepatocytes and thereby limit stellate cell activation and the associated increases in collagen synthesis. First, despite similar levels of hepatocyte injury (S3 Fig), scAAV8.eGFP.miR29a treatment was associated with reduced immunostaining for α-SMA (S4 Fig), a well-described marker of activated stellate cells and myofibroblasts. Second, miR-29 is detectably expressed in normal hepatocytes and we observed that exposure of these cells to the profibrotic cytokine TGF-β decreased expression of miR-29a/b/c (Fig 1D). Third, the importance of maintaining normal miR-29 expression in hepatocytes is highlighted by a previous report which showed that hepatocyte specific knockout of miR-29 was associated with increased susceptibility to liver fibrosis [28]. In addition to altering hepatocyte mRNA expression profiles, non-cell autonomous effects of transgene-derived miR-29a could also contribute to the observed anti-fibrotic benefits of scAAV8.eGFP.miR29a treatment. In this scenario, transgene derived miR-29a would transit from hepatocytes to neighboring, non-transduced stellate cells where it could post-transcriptionally repress collagen and other ECM protein expression. In support of this possibility, the transfer of functional miRNAs, including miR-29, to other cells via gap junctions or exosomes has been described [33–38]. Finally, the anti-fibrotic benefits of scAAV8.eGFP.miR29a may reflect not only decreased ECM synthesis but could also involve increased matrix metabolism associated with altered expression of matrix metalloproteinases (MMPs) and/or tissue inhibitor of metalloproteinases (TIMPs).

Independent of the specific mechanism of action, stable transgene expression above the therapeutic threshold is essential for long-term protection and we assessed AAV durability in our models by quantifying viral genomes, GFP+ cells and miR-29a levels. We found that high levels of both scAAV8.eGFP and scAAV8.miR29a.eGFP are present in uninjured mice four weeks after injection (Fig 2B). Despite the persistence of scAAV8.miR29a.eGFP, a single injection of either 2x10^11^ or 1x10^12^ viral genomes was not sufficient to increase hepatic miR-29a expression above normal levels in uninjured mice (S2 Fig). Importantly, this does not appear to be an inherent limitation of the scAAV8.miR-29a.eGFP construct as in vitro transfection with increasing amounts of this plasmid is associated with concordant increases in miR-29a (S2 Fig). It is also in contrast to a previous report that mice transduced with 1x10^12^ scAAV8.miR26a.eGFP genomes exhibited significant overexpression of miR-26a in liver [25]. Together, these observations suggest that the in vivo maturation of miR-29a is tightly regulated under normal physiologic conditions and thus, in the absence of an injury or other external stimuli, the addition of virally produced precursor transcripts will not result in a net increase of mature miR-29a. While such tight regulation of miR-29 production could limit the utility of this approach in settings where supraphysiologic miRNA levels are required to reach the therapeutic threshold, it also provides natural protection against potential toxicity from virally-derived miR-29 overexpression.

Liver injury can rapidly dilute AAV vector genomes [27] and after 4 weeks of CCl4 treatment, scAAV8.miR-29a.eGFP genomes (Fig 3B) were reduced compared to untreated (no CCl_4_) mice. Importantly though, the residual genomes were sufficient to maintain normal miR-29a levels (Fig 3C) and it therefore appears that processing of virally-derived miR-29 transcripts can counteract the decrease in endogenous miR-29 levels that otherwise occurs in the setting of chronic liver injury. In contrast to the relative stability of scAAV8.miR-29a.eGFP, four weeks of CCl_4_ treatment dramatically reduces scAAV8.eGFP genomes to near undetectable levels. GFP immunofluorescence was detectable after four weeks of CCl_4_ in a similarly high percentage of hepatocytes in both scAAV8.eGFP and scAAV8.miR-29a.eGFP treated animals (S5 Fig), indicating that GFP protein remains detectable for some time after the loss of viral genomes. After 8 weeks of CCl4 treatment, viral genomes are nearly undetectable in both scAAV8.eGFP and scAAV8.miR-29a.eGFP injected mice and miR-29a levels are reduced in both cohorts compared to untreated (no CCl4) mice (Fig 4). The fact that GFP is still detectable in a large number of hepatocytes 8 weeks after scAAV8.miR-29a.eGFP injection (S5 Fig) suggests the loss of viral genomes was a relatively recent occurrence and thus viral miR-29a expression was present for most of the period of CCl_4_ exposure. Together, our findings demonstrate that under conditions of ongoing CCl_4_-mediated liver injury, a single injection of 2x10^11^ scAAV8.miR-29a.eGFP genomes is sufficient to normalize hepatic miR-29a expression for more than 4 weeks but less than 8 weeks.

In summary, we demonstrate here that a single injection of scAAV8.miR29a.eGFP ameliorates fibrosis when administered prior to or after the onset of liver injury. While elucidation of specific therapeutic mechanisms and further refinement of delivery methods will aid ongoing efforts to develop clinically viable strategies, the antifibrotic protection associated with parenchymal transgene expression suggests that therapeutic miR-29 delivery may be effective in treating a variety of fibroproliferative disorders.

## Supporting Information

S1 Fig *COL1A1* mRNA is a potent miR-29a target. (**a**) Alignment of the 3' UTRs of the *COL1A1* gene from various species showing three highly conserved miR-29 target sites. The mutations created in each of the miR-29 target sites in the luciferase reporter construct used in **b** are shown in red. (**b**) Relative firefly luciferase activity from WT and mutant (Mut) human *COL1A1* 3' UTR reporter constructs following transfection into primary human fibroblasts with or without control or miR-29 antisense (AS) oligonucleotides. *Renilla* luciferase activity produced from a co-transfected control plasmid allowed for normalization of transfection efficiency. (**c**) Western blot showing increased type I collagen protein in primary human fibroblasts transfected with miR-29 antisense oligonucleotides.
(PDF)

S2 Fig *In vitro* and *in vivo* miR-29 expression levels associated with AAV.miR-29.eGFP. **(a)** miR-29a expression in HEK293 cells transfected with varying amounts of AAV.eGFP or AAV.miR-29a.eGFP plasmids. Fold change was calculated using mock transfected (0ng) cells as a control. **(b)** Hepatic mir-29a expression in mice receiving a single injection of low dose (2x10^11^ vg) or high dose (1x10^12^ vg) AAV. Average fold change was calculated using normal (no AAV) as a control. Error bars represent +/- one standard deviation.
(PDF)

S3 Fig AST and ALT serum levels in scAAV8.eGFP and scAAV8.miR29.eGFP treated mice. (PDF)

S4 Fig α-SMA expression in scAAV8.eGFP and scAAV8.miR29.eGFP treated mice. Sections of transduced livers were immunostained for α-SMA. Representative sections from scAAV8.eGFP and scAAV8.miR29.eGFP treated mice after four weeks and 12 weeks of CCl_4_ treatment are shown. Scale bar = 100μm.
(PDF)

S5 Fig Percent GFP+ cells in liver samples of scAAV8.eGFP and scAAV8.miR29.eGFP treated mice. Liver samples from each mouse were immunostained for eGFP and counterstained with DAPI. For each sample, the number of eGFP positive cells and Hoechst-positive nuclei were determined in four independent fields using a 40x objective. The percent GFP+ cells across the four windows was averaged for each mouse and the graph shows the mean GFP+ cells (+/- 1 standard deviation) for each cohort.
(PDF)

S1 Methods Descriptive Methods for Supplemental Figures. (PDF)
