# Supplementary figures and images for: Systemic Delivery of scAAV8-Encoded MiR-29a Ameliorates Hepatic Fibrosis in Carbon Tetrachloride-Treated Mice
Source: PLoS One. 2015 Apr 29;10(4):e0124411. doi: 10.1371/journal.pone.0124411 (PMC4414421; doi:10.1371/journal.pone.0124411)

S1 Fig.

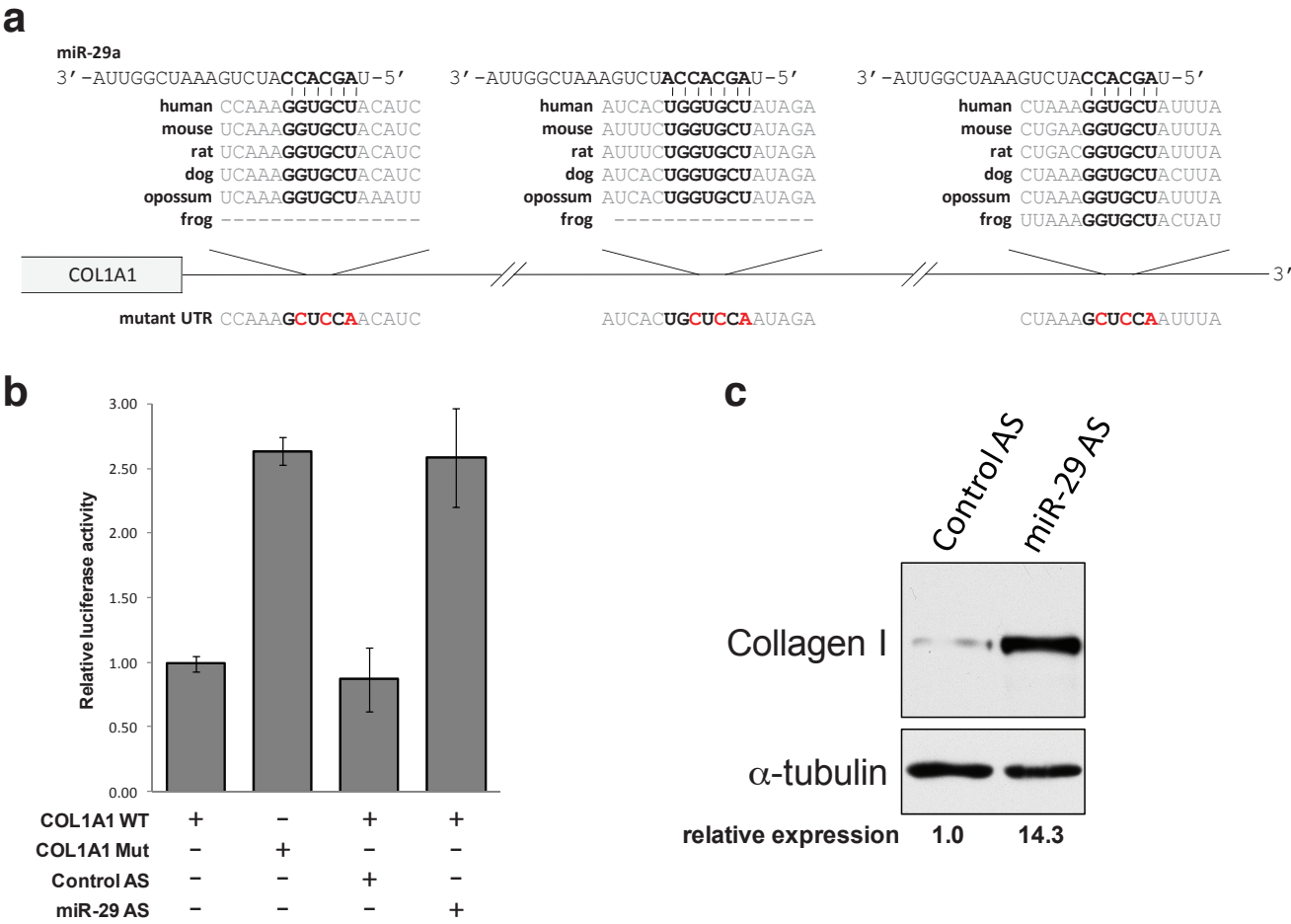

Supplement: S1 Fig — (a) Alignment of the 3' UTRs of the COL1A1 gene from various species showing three highly conserved miR-29 target sites. The mutations created in each of the miR-29 target sites in the luciferase reporter construct used in b are shown in red. (b) Relative firefly luciferase activity from WT and mutant (Mut) human COL1A1 3' UTR reporter constructs following transfection into primary human fibroblasts with or without control or miR-29 antisense (AS) oligonucleotides. Renilla luciferase activity produced from a co-transfected control plasmid allowed for normalization of transfection efficiency. (c) Western blot showing increased type I collagen protein in primary human fibroblasts transfected with miR-29 antisense oligonucleotides. (PDF) [file pone.0124411.s001.pdf]

S2 Fig.

a

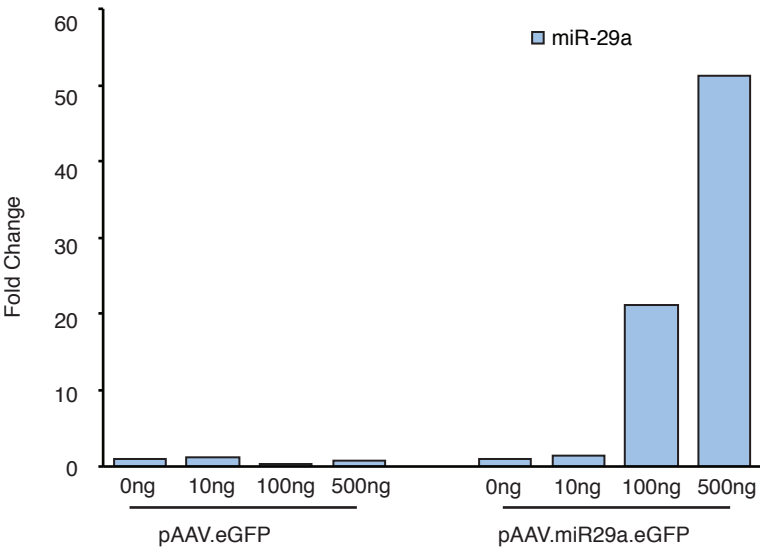

b

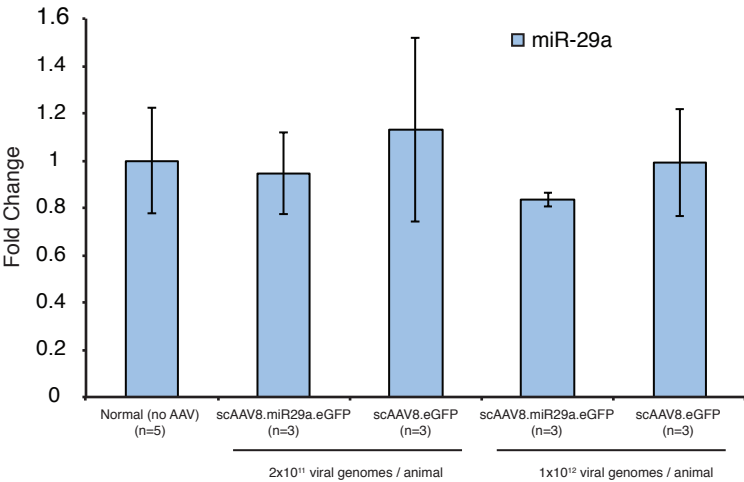

Supplement: S2 Fig — (a) miR-29a expression in HEK293 cells transfected with varying amounts of AAV.eGFP or AAV.miR-29a.eGFP plasmids. Fold change was calculated using mock transfected (0ng) cells as a control. (b) Hepatic mir-29a expression in mice receiving a single injection of low dose (2x10^11^ vg) or high dose (1x10^12^ vg) AAV. Average fold change was calculated using normal (no AAV) as a control. Error bars represent +/- one standard deviation. (PDF) [file pone.0124411.s002.pdf]

S3 Fig.

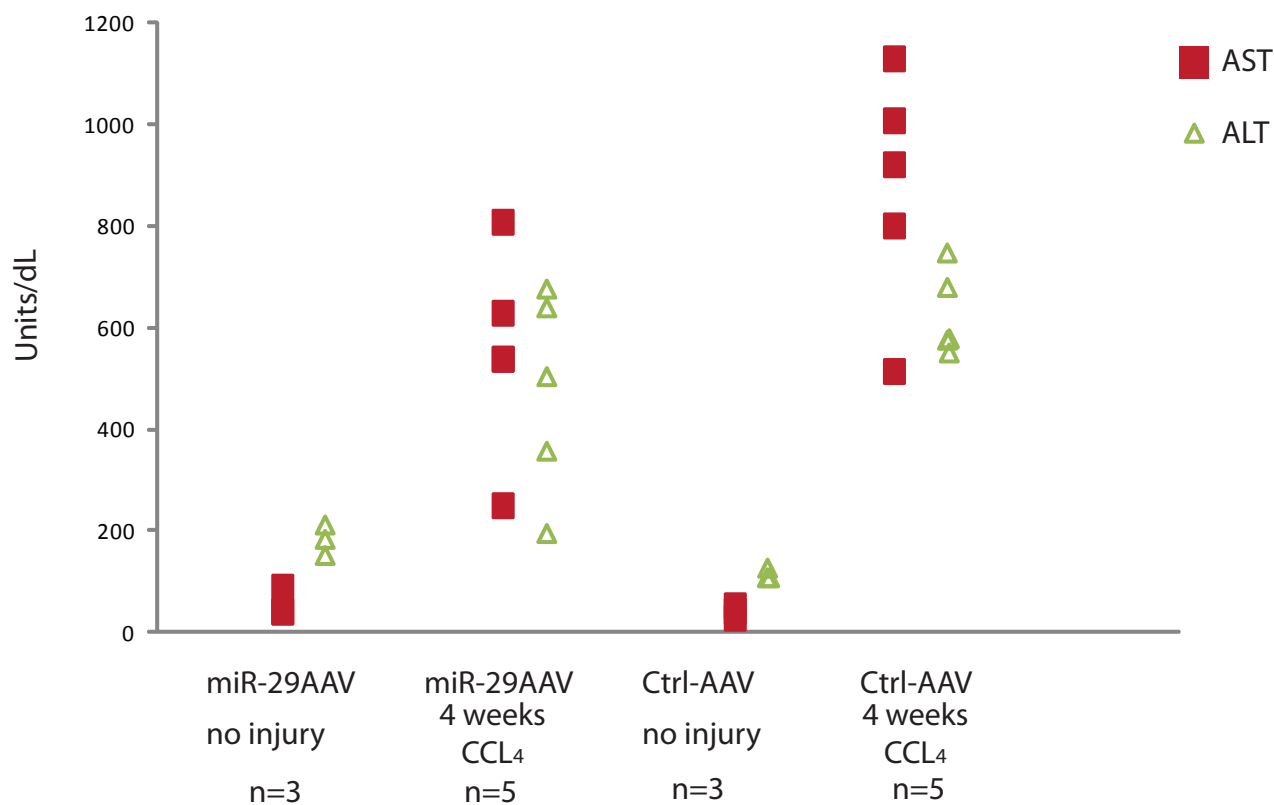

Supplement: S3 Fig — (PDF) [file pone.0124411.s003.pdf]

S4 Fig.

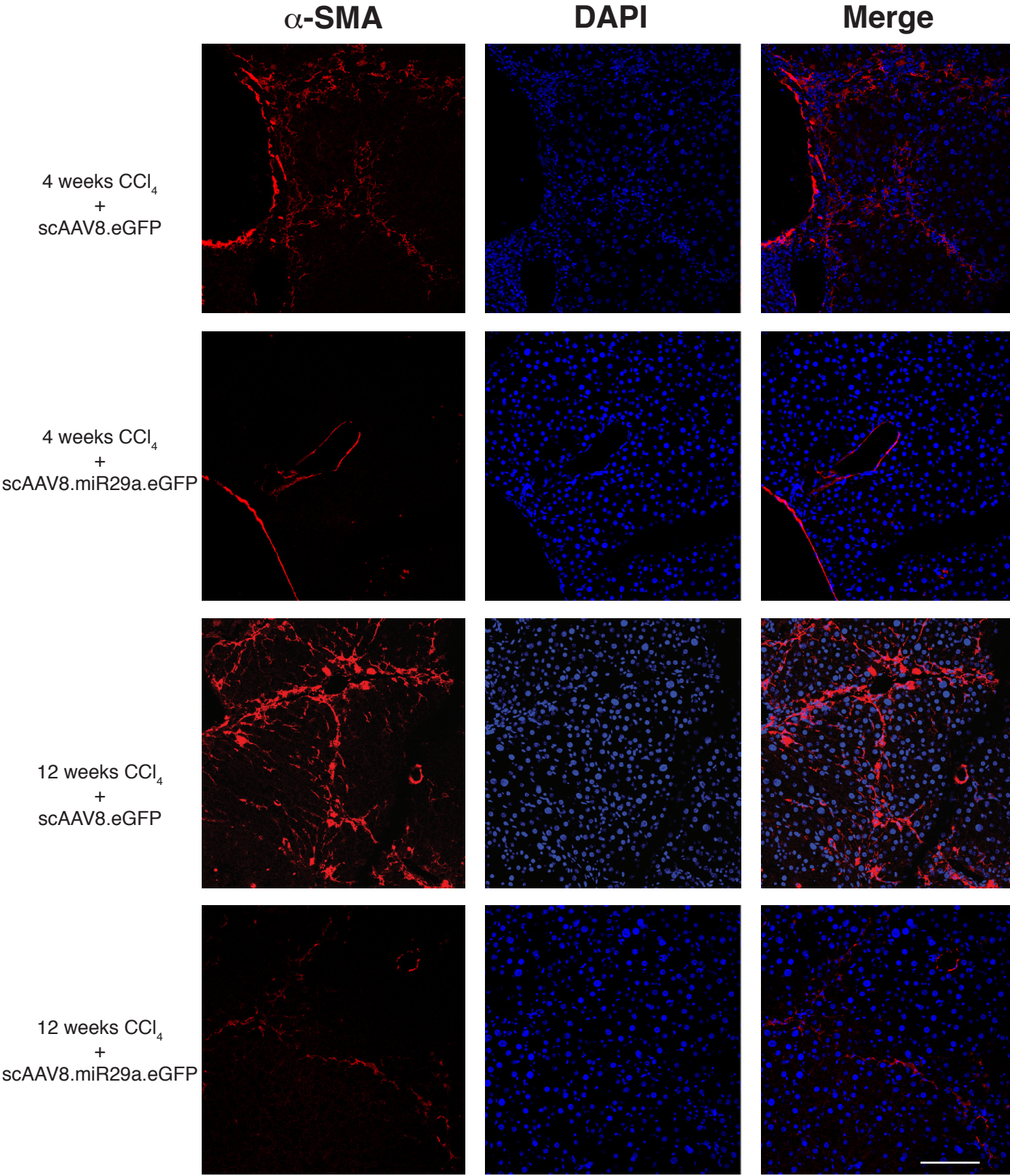

Supplement: S4 Fig — Sections of transduced livers were immunostained for α-SMA. Representative sections from scAAV8.eGFP and scAAV8.miR29.eGFP treated mice after four weeks and 12 weeks of CCl4 treatment are shown. Scale bar = 100μm. (PDF) [file pone.0124411.s004.pdf]

S5 Fig.

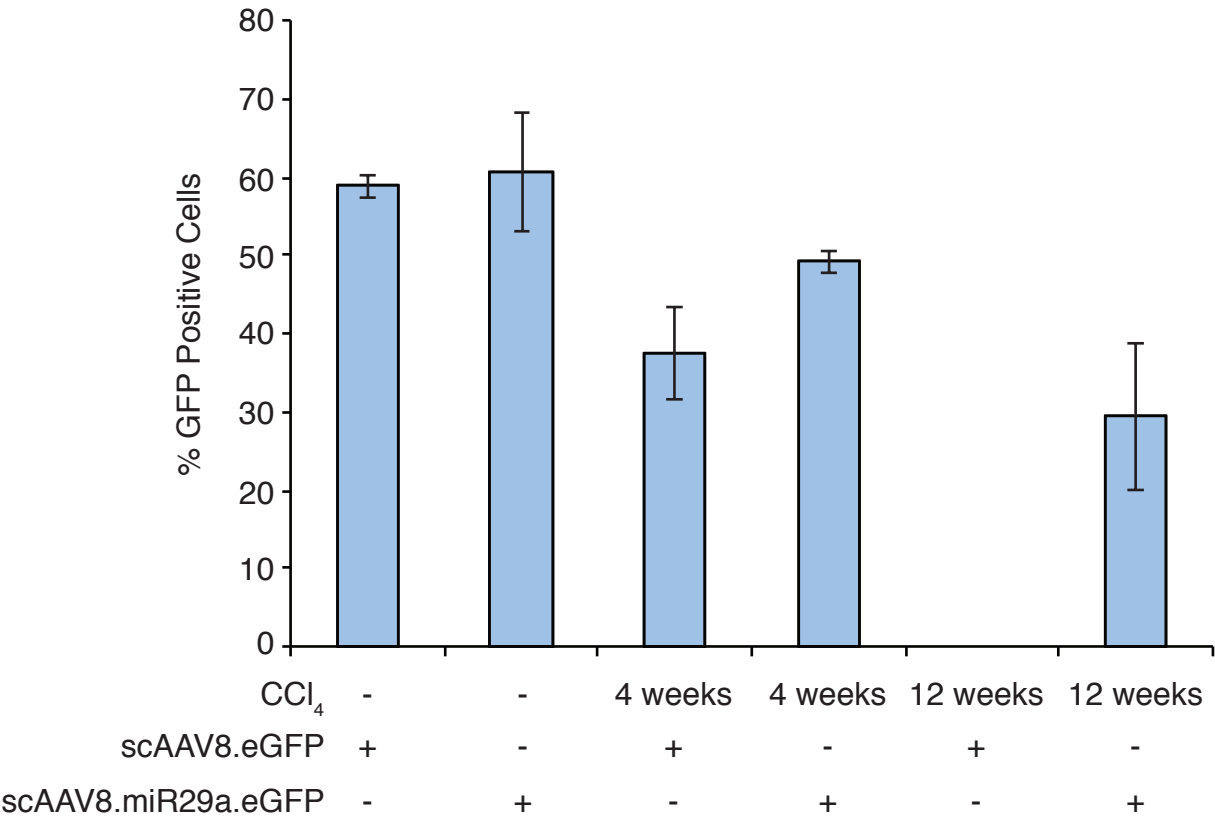

Supplement: S5 Fig — Liver samples from each mouse were immunostained for eGFP and counterstained with DAPI. For each sample, the number of eGFP positive cells and Hoechst-positive nuclei were determined in four independent fields using a 40x objective. The percent GFP+ cells across the four windows was averaged for each mouse and the graph shows the mean GFP+ cells (+/- 1 standard deviation) for each cohort. (PDF) [file pone.0124411.s005.pdf]
