# Supplementary material for: Systemic Delivery of scAAV8-Encoded MiR-29a Ameliorates Hepatic Fibrosis in Carbon Tetrachloride-Treated Mice
Source: PLoS One. 2015 Apr 29;10(4):e0124411. doi: 10.1371/journal.pone.0124411 (PMC4414421; doi:10.1371/journal.pone.0124411)
Supplement: S1 Methods — (PDF) [file pone.0124411.s006.pdf]

S1 Methods

Luciferase reporter assays

The human *COL1A1* 3' UTR luciferase reporter plasmid was constructed by PCR amplifying a 534 bp fragment containing the predicted miR-29 recognition sites from human genomic DNA using the following primers: 5'-ATACCGTCTAGAAACGCGTGTCAATCCCTTGTG-3' (forward); 5'-ATACCGTCTAGAGGACAGAGGACGCAGGACAGA-3' (reverse). The amplicon was digested and inserted into the XbaI site of pGL3-control (Promega). Mutagenesis was performed using the Quikchange XL kit (Stratagene) according to the manufacturer's instructions. 24 hours prior to transfection, low-passage human primary fibroblasts were plated at 25,000 per well of a 24-well plate. 100 ng of *COL1A1* reporter plasmid and 50 ng of renilla luciferase control plasmid (phRL-SV40, from Promega) with or without miR-29 inhibitor were transfected using lipofectamine 2000 (Invitrogen) according to the manufacturer's instructions. Control and miR-29 inhibitors were obtained from Dharmacon. 10 nM each of miR-29a, -29b, and -29c inhibitors were included in the transfection mix to achieve 30 nM final concentration and the control inhibitor was used at 30 nM. Luciferase assays were performed 24 hours after transfection using the Dual Luciferase Reporter Assay System (Promega). Each transfected well was assayed in triplicate. Firefly luciferase activity was normalized to Renilla luciferase activity for each transfected well.

Western Blot

Low-passage human foreskin primary fibroblasts (obtained from ATCC, CRL-2091) were transfected with control or miR-29 inhibitors (Dharmacon) using DharmaFECT1 according to the manufacturer's instructions. 10 nM each of miR-29a, -29b, and -29c inhibitors were included in the transfection mix to achieve 30 nM final concentration and the control inhibitor was used at 30 nM. Immunoblotting was performed with anti-collagen I (Abcam, ab292) and anti-α-tubulin mouse monoclonal (clone DM1A; Calbiochem).
